# Prognostic and predictive value of tumor infiltration proportion within lymph nodes in N1 colorectal cancer

**DOI:** 10.3389/fonc.2025.1512960

**Published:** 2025-03-25

**Authors:** Rujie Chen, Jun Zhu, Dong Xu, Xiaoyan Fan, Yihuan Qiao, Xunliang Jiang, Jun Hao, Yongtao Du, Xihao Chen, Guo Yuan, Jipeng Li

**Affiliations:** ^1^ Department of Digestive Surgery, Xijing Hospital of Digestive Diseases, Fourth Military Medical University, Xi’an, China; ^2^ Department of General Surgery, The Southern Theater Air Force Hospital, Guangzhou, China; ^3^ Division of Digestive Surgery, Xi’an International Medical Center Hospital of Digestive Diseases, Xi’an, China; ^4^ Department of Experiment Surgery, Xijing Hospital, Fourth Military Medical University, Xi’an, China; ^5^ Department of Breast, Shaanxi Provincial Cancer Hospital, Xi’an, China

**Keywords:** colorectal cancer, N1 stage, tumor infiltration proportion, lymph nodes, prognostic model

## Abstract

**Introduction:**

Lymph node metastasis is a crucial determinant of prognosis in colorectal cancer (CRC), significantly impacting survival outcomes and treatment decision-making. This study aims to evaluate the prognostic value of tumor infiltration proportion within lymph nodes (TIPLN) in N1 CRC patients and to develop a TIPLN-based nomogram to predict prognosis.

**Methods:**

A total of 416 N1 CRC patients who underwent radical resection were enrolled and divided into training and validation cohorts. Whole-slide images of lymph nodes were annotated to assess the TIPLN. Univariable and multivariable Cox regression analyses were conducted to identify independent prognostic factors and to develop a nomogram for predicting patient outcomes. The precision and discrimination of the nomogram were evaluated using the area under the receiver operating characteristic curve (AUC), concordance index (C-index), and calibration curve. Decision curve analysis (DCA) was performed to compare the net benefit of the nomogram at different threshold probabilities. Additionally, net reclassification index (NRI) and integrated discrimination improvement (IDI) were used to evaluate the nomogram’s clinical utility.

**Results:**

High TIPLN levels were significantly associated with poorer overall survival (OS). Five variables, including TIPLN, were selected to construct the nomogram. The C-index in OS prediction was 0.739 and 0.753 for the training and validation cohorts, respectively. Additionally, strong precision and discrimination were demonstrated through AUC and calibration curves. The NRI (training cohort: 0.191 for 3-year and 0.436 for 5-year OS prediction; validation cohort: 0.180 for 3-year and 0.439 for 5-year OS prediction) and IDI (training cohort: 0.079 for 3-year and 0.094 for 5-year OS prediction; validation cohort: 0.078 for 3-year and 0.098 for 5-year OS prediction) suggest that the TIPLN-based nomogram significantly outperformed the clinicopathological nomogram. Furthermore, DCA demonstrated the high clinical applicability of the TIPLN-based nomogram for predicting OS.

**Conclusions:**

TIPLN could serve as a prognostic predictor for N1 CRC patients. The TIPLN-based nomogram enhances survival prediction accuracy and facilitates more informed, individualized clinical decision-making.

## Introduction

Despite significant advancements in screening and treatment, colorectal cancer (CRC) remains an important contributor to the global burden of cancer (1). The 5-year overall survival (OS) rate ranges from 92% for patients diagnosed at stage I to as low as 11% for those diagnosed at stage IV (2, 3).

The American Joint Committee on Cancer (AJCC) TNM staging system is widely adopted to stratify CRC patients and guide treatment decisions (4), relying on tumor size (T), lymph node involvement (N), and the presence of distant metastasis (M) (5). Among these factors, lymph node (LN) involvement plays a particularly crucial role in determining prognosis (6, 7). Patients with LN metastasis are classified at least as stage III regardless of the depth of tumor penetration, which significantly affects survival outcomes (8).

However, the current AJCC-N staging (8), which is based primarily on the number of positive LNs, presents several limitations. Survival rates among patients within the same N stage often differ considerably (9–11), indicating significant biological heterogeneity that the current classification fails to adequately capture. To refine prognostic assessment, additional metrics such as the lymph node ratio (LNR) (12, 13) and the log odds of positive lymph nodes (LODDS) (14, 15) have been proposed, incorporating both the number of positive nodes and the total number of lymph nodes examined (Examined N). Nevertheless, while both LNR and LODDS offer valuable insights into disease assessment (16, 17), their predictive utility has not yet consistently demonstrated a significant advantage over the conventional N staging system (18, 19). These metrics remain largely focused on quantitative aspects of nodal involvement, potentially limiting their ability to fully reflect the biological complexity of tumor progression.

A recent study from Wang et al. introduced a novel prognostic indicator for gastric cancer by calculating the ratio of tumor cell area to lymph node area (20). Their findings indicated that the tumor cell area ratio progressively increased with disease stage, and higher ratios were significantly associated with poorer prognosis. These results highlight the importance of considering LN characteristics beyond node count. However, the role of the tumor infiltration proportion within lymph nodes (TIPLN) in CRC has not yet been investigated.

Thus, the present study aims to assess the prognostic value of TIPLN in patients with CRC. Additionally, we seek to develop and validate a nomogram incorporating TIPLN and other clinical variables to provide a precise tool for prognostic assessment in CRC patients.

## Materials and methods

### Study design and patients

**Figure 1:**
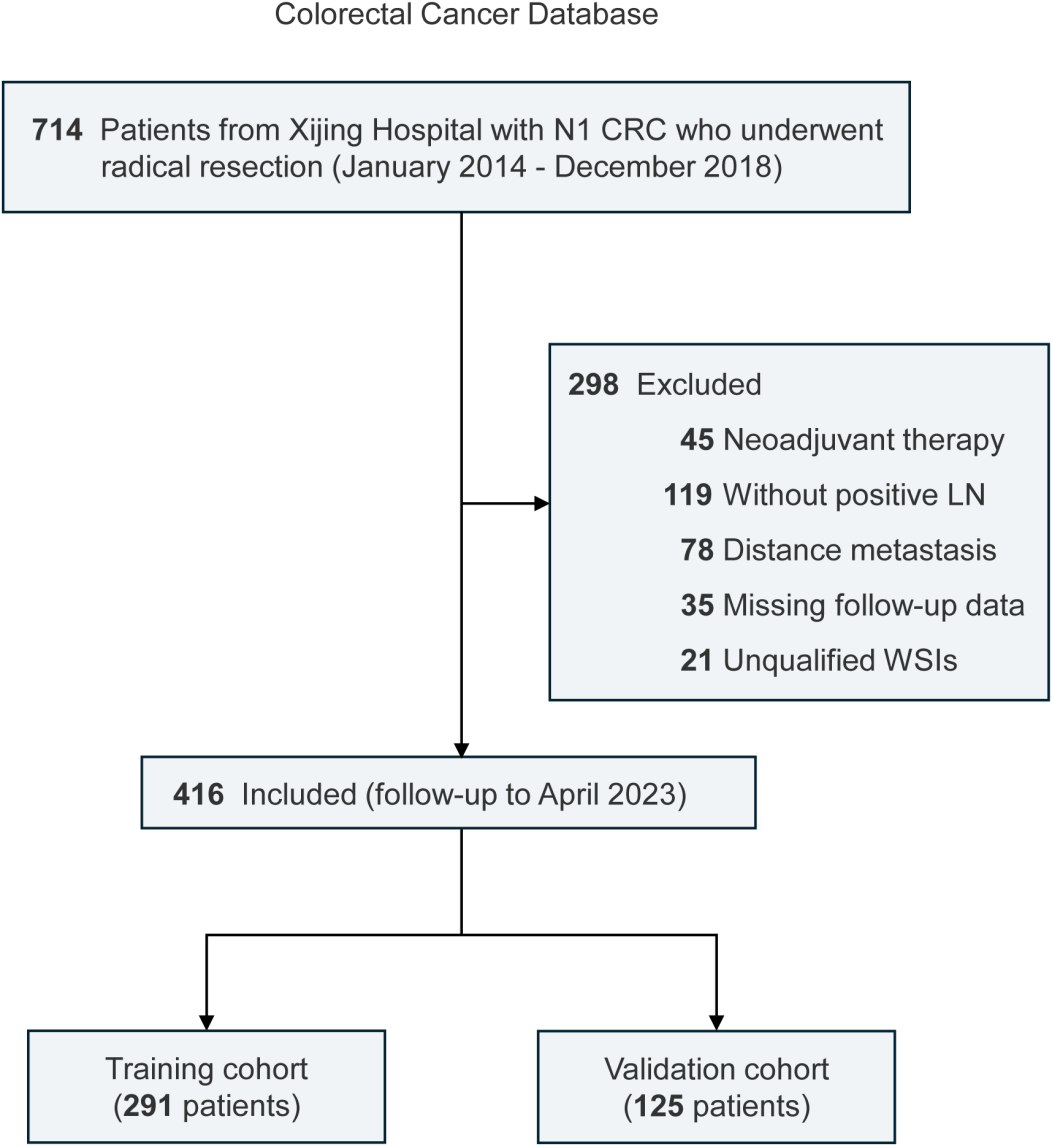
Flowchart and selection process of the study.

According to the 8th edition of the AJCC TNM staging system (8), N1a (1 positive LN) and N1b (2-3 positive LNs) CRC are classified under the same prognostic category. To ensure cohort homogeneity and address technical challenges in quantifying TIPLN across multiple LNs, we restricted our analysis to N1 patients. This approach minimized variability from extensive LN involvement and facilitated reliable TIPLN measurement. This retrospective study included N1 CRC patients who underwent radical resection at Xijing Hospital from January 2014 to December 2018. The inclusion criteria were as follows: (i) pathologically confirmed N1 CRC; (ii) underwent radical surgical resection; (iii) complete clinicopathological data. The exclusion criteria were: (i) receipt of neoadjuvant therapy; (ii) absence of positive LNs; (iii) presence of distant metastasis at diagnosis; (iv) incomplete follow-up data; (v) unqualified pathological slides. The flowchart outlining the selection process of the study is shown in **Figure 1**. Finally, 416 patients with 713 pathological slides were included. The patients were subsequently divided into training and validation cohorts in a 7:3 ratio. The analysis followed the Strengthening the Reporting of Observational Studies in Epidemiology (STROCSS) guidelines (21).

### Data collection

The baseline clinicopathological data were obtained from electronic medical records, comprising patient demographics (age and sex), pathological features of surgical specimens (tumor size, T stage, N stage, and Examined N), tumor biomarkers (carcinoembryonic antigen [CEA], carbohydrate antigen 19-9 [CA19-9], and carbohydrate antigen 125 [CA125]), as well as follow-up data. Elevated biomarker levels were defined as follows: CEA > 5 ng/mL, CA125 > 35 U/mL, and CA19-9 > 37 U/mL. OS was selected as the primary endpoint, which was calculated from the time of surgery until death of any cause or last follow-up.

### Ethics statement

The study protocol was approved by the Medical Ethics Committee of the First Affiliated Hospital of the Air Force Medical University with a waiver for informed consent (KY20212211-C-1).

### Evaluation of TIPLN

The TIPLN for each positive LN was defined as the ratio of the tumor area to the total area of the LN. All hematoxylin and eosin (H&E)-stained pathological slides of lymph nodes were retrieved from the pathology archive and scanned into whole slide images (WSIs) using the Olympus VS200 slide scanner at 20× magnification. The WSIs were independently analyzed by two experienced pathologists, who were blinded to the clinical data of the patients. Tumor and LN regions were carefully annotated using distinct colors with the OLYMPUS OlyVIA 3.3 software.

For cases where the TIPLN values determined by the two pathologists differed by less than 10%, the average of their measurements was used. If the discrepancy in TIPLN values exceeded 10%, a third senior pathologist reviewed the WSIs, and a consensus was reached through discussion.

Both the average and maximum TIPLN values across all positive LNs were initially considered. However, for the final analysis, the maximum TIPLN value for each patient was used, as it demonstrated a stronger prognostic association in preliminary survival analyses, reflected by a higher hazard ratio (HR).

### Development and assessment of the nomogram

In the training cohort, univariate Cox regression was used to evaluate the association of TIPLN and clinical characteristics with OS. Independent prognostic factors were identified using multivariate Cox regression (entry criterion: *P* < 0.05) with backward stepwise selection, applying Akaike’s information criterion (AIC) as the stopping rule. These factors were then incorporated into the nomogram for predicting 3- and 5-year OS.

The nomogram assigns points to each covariate based on its contribution to prognosis, with the variable having the highest beta coefficient receiving 100 points. Total points are summed, and the predicted OS probability is determined by drawing a vertical line from the total points axis.

The nomogram’s performance was assessed using the area under the receiver operating characteristic (ROC) curve (AUC) and the concordance index (C-index). Calibration curves, based on 1000 bootstrap resamples, were generated to evaluate the alignment between observed outcomes and the nomogram’s predictions. The net reclassification index (NRI), integrated discrimination improvement (IDI), and decision curve analysis (DCA) were used to compare the performance of different prognostic models (22–24). NRI and IDI evaluated improvements in classification and discrimination for 3- and 5-year OS. DCA evaluated clinical utility by measuring the net benefit across various threshold probabilities, identifying individuals with sufficiently high risk to recommend for intervention or treatment. The validation cohort was used to confirm model discrimination, calibration, and clinical utility (25).

### Statistical analysis

Descriptive statistics are reported as frequencies for categorical variables and medians and interquartile ranges (IQR) for continuous variables. Continuous variables were compared using an independent-sample, unpaired two-tailed t test or Mann−Whitney H test, as appropriate. Differences in categorical variables were compared using the chi-square or Fisher’s exact test. The Kaplan-Meier curve was plotted and log rank (Mantel-Cox) test was applied to evaluate OS differences. Cox regression analysis was used for univariate and multivariate analyses, and the HR with 95% CI was calculated. All statistical analyses were performed using R (version 4.2.1) software. All tests were two-sided; *P* < 0.05 was considered significant.

## Results

### Patient characteristics

**Table 1** presents the clinicopathological characteristics of patients in the training (n = 291) and validation (n = 125) cohorts. Of the 416 participants, 235 (56.5%) patients were male, and the median age was 62 (IQR 53-69) years. The majority of patients (70.7%, 294/416) were diagnosed with stage T3. The median (IQR) follow-up duration in the training cohort was 60 (53–66) months, with 3-year and 5-year OS rates of 83.3% and 74.3%, respectively. In the validation cohort, the median (IQR) follow-up duration was 59 (51-68) months, with 3-year and 5-year OS rates of 80.8% and 70.4%, respectively. No significant differences were observed between the two cohorts in demographic and clinical characteristics (*P* > 0.05).

**Table 1:** Characteristics of the participants in the training and validation cohorts.

Characteristic^a^	Whole population N = 416	Training cohort N = 291	Validation cohort N = 125^1^	P
Age, years	62 (53-69)	62 (54-69)	61 (52-67)	0.260
Sex				0.933
Male	235 (56.5%)	164 (56.4%)	71 (56.8%)	
Female	181 (43.5%)	127 (43.6%)	54 (43.2%)	
T stage				0.926
T1	8 (1.9%)	6 (2.1%)	2 (1.6%)	
T2	58 (13.9%)	39 (13.4%)	19 (15.2%)	
T3	294 (70.7%)	208 (71.5%)	86 (68.8%)	
T4	56 (13.5%)	38 (13.1%)	18 (14.4%)	
N stage				0.369
N1a	197 (47.4%)	142 (48.8%)	55 (44.0%)	
N1b	219 (52.6%)	149 (51.2%)	70 (56.0%)	
Size, no. (%)				0.523
≤ 5 (cm)	287 (69.0%)	198 (68.0%)	89 (71.2%)	
> 5 (cm)	129 (31.0%)	93 (32.0%)	36 (28.8%)	
Examined N	16.0 (13.0-19.0)	16.0 (13.0-19.0)	15.0 (12.0-19.0)	0.447
Albumin, (g/L)				0.687
≥ 40	157 (37.7%)	108 (37.1%)	49 (39.2%)	
< 40	259 (62.3%)	183 (62.9%)	76 (60.8%)	
CEA, ng/mL				0.970
≤ 5	329 (79.1%)	230 (79.0%)	99 (79.2%)	
> 5	87 (20.9%)	61 (21.0%)	26 (20.8%)	
CA19-9, U/mL				0.625
≤ 37	354 (85.1%)	246 (84.5%)	108 (86.4%)	
> 37	62 (14.9%)	45 (15.5%)	17 (13.6%)	
CA125, U/mL				0.826
≤ 35	391 (94.0%)	274 (94.2%)	117 (93.6%)	
> 35	25 (6.0%)	17 (5.8%)	8 (6.4%)	

a Values are presented as median (IQR) for continuous variables and as count (percentage) for categorical variables.

Examined N, total number of lymph nodes examined; CEA, carcinoembryonic antigen; CA19-9, carbohydrate antigen19-9; CA125, carbohydrate antigen125.

### High TIPLN levels predict poor prognosis in CRC patients

**Figure 2:**
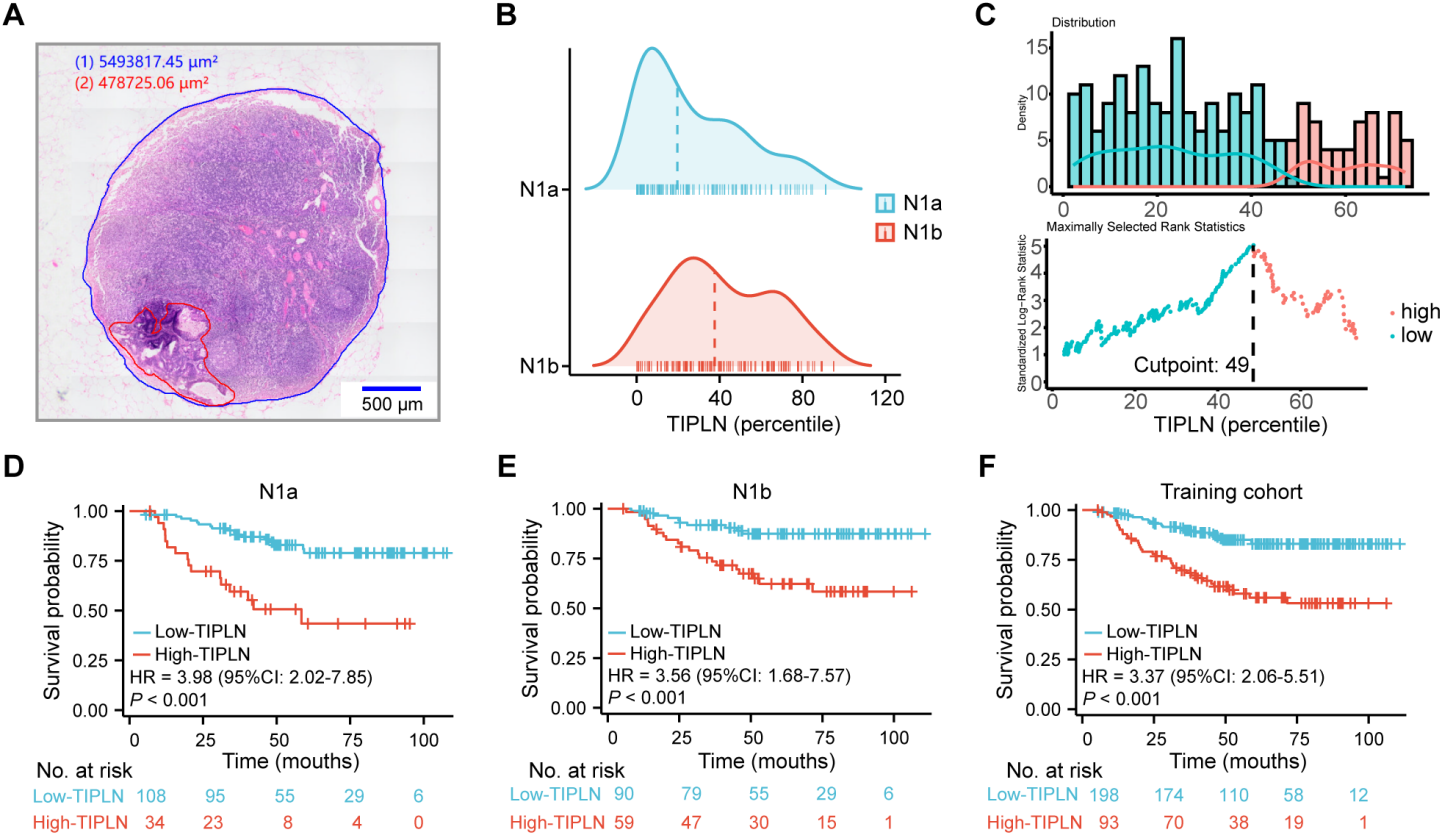
Association of TIPLN and overall risk of death in N1 patients. **(A)** Representative image of the annotated WSIs for lymph node quantification (red for tumor tissue, and blue for lymph node tissue). **(B)** Distribution of TIPLN in N1a and N1b patients, with the dashed lines indicating median. **(C)** Selection of the optimum cutoff value for the TIPLN. Histogram shows the density distribution for high- and low-TIPLN groups divided by the optimum cutoff value, while the scatter plot displays the standardized log-rank statistic value for each TIPLN cutoff value. **(D)** Kaplan-Meier curve for N1a patients. **(E)** Kaplan-Meier curve for N1b patients. **(F)** Kaplan-Meier curve for the entire training cohort. TIPLN, tumor infiltration proportion within lymph nodes; HR, hazard ratio; CI, confidence interval.

As illustrated in **Figure 2A**, a representative pathological slide shows the LN region marked in blue and the tumor region in red, facilitating the calculation of the TIPLN. The distribution of TIPLN values among N1a and N1b patients in the training cohort is shown in **Figure 2B**, with median TIPLN values of 19% (IQR 5%-46%) for N1a patients and 38% (IQR 23%-63%) for N1b patients.

In the training cohort, a cut-off value of 49%, determined through maximally selected log-rank statistics (26), was used to distinguish between high and low TIPLN groups (**Figure 2C**). Kaplan-Meier curves (**Figures 2D–F**) demonstrate that patients with high-TIPLN had significantly worse OS compared to those with low-TIPLN. Consistent trends were observed across subgroup analyses within the training cohort: N1a (HR = 3.98, 95% CI: 2.02-7.85, *P* < 0.001), N1b (HR = 3.56, 95% CI: 1.68-7.57, *P* < 0.001), and the overall cohort (HR = 3.37, 95% CI: 2.06-5.51, *P* < 0.001). Similar survival trends were observed in the validation cohort (**Supplementary Figure 1**), further strengthening the evidence that TIPLN provides additional prognostic value beyond conventional N staging in CRC. These findings validate the robustness and generalizability of our results.

### Development and validation of the nomogram for prognosis

In the univariate Cox regression analysis, T stage, Examined N, albumin, CEA, CA19-9, CA125, and TIPLN were significantly associated with OS in the training cohort. TIPLN was still positively associated with worse OS in multivariable Cox regression analysis after adjusting for T stage, Examined N, albumin, CEA, CA19-9, and CA125 (HR = 2.66, 95% CI: 1.58-4.47, *P* < 0.001; **Table 2**).

**Table 2:** Univariate and multivariate analyses of overall survival in the training cohort.

Variable	Univariate analysis		Multivariate analysis	
	HR (95% CI)	*P*	HR (95% CI)	*P*
Age			–	–
≤60 years	Reference			
>60 years	1.10 (0.67-1.79)	0.701		
Sex			–	–
Male	Reference			
Female	1.12 (0.69-1.82)	0.654		
T stage				
T1-2	Reference		Reference	
T3-4	2.54 (1.10-5.89)	0.029	2.71 (1.16-6.33)	0.023
N stage				
N1a	Reference			
N1b	1.22 (0.75-2.00)	0.413		
Tumor Size				
≤5 cm	Reference		–	–
>5 cm	1.31 (0.79-2.17)	0.289	–	–
Examined N				
≥12	Reference		Reference	
<12	1.79 (1.02-3.15)	0.042	1.60 (0.90-2.84)	0.112
TIPLN				
Low	Reference		Reference	
High	3.37 (2.06-5.51)	<0.001	2.66 (1.58-4.47)	<0.001
Albumin				
≥40 g/L	Reference		Reference	
<40 g/L	2.36 (1.45-3.85)	<0.001	2.19 (1.33-3.58)	0.002
CEA				
≤5 ng/mL	Reference		Reference	
>5 ng/mL	2.80 (1.70-4.60)	<0.001	2.09 (1.19-3.69)	0.010
CA19-9				
≤37 U/mL	Reference		Reference	
>37 U/mL	3.07 (1.81-5.21)	<0.001	1.29 (0.69-2.40)	0.428
CA125				
≤35 U/mL	Reference		Reference	
>35 U/mL	2.63 (1.26-5.53)	0.010	1.51 (0.69-3.29)	0.304

HR, hazard ratio; CI, confidence interval. Examined N, total number of lymph nodes examined; TIPLN, tumor invasion proportion of lymph nodes; CEA, carcinoembryonic antigen; CA19-9, carbohydrate antigen19-9; CA125, carbohydrate antigen125.

**Figure 3:**
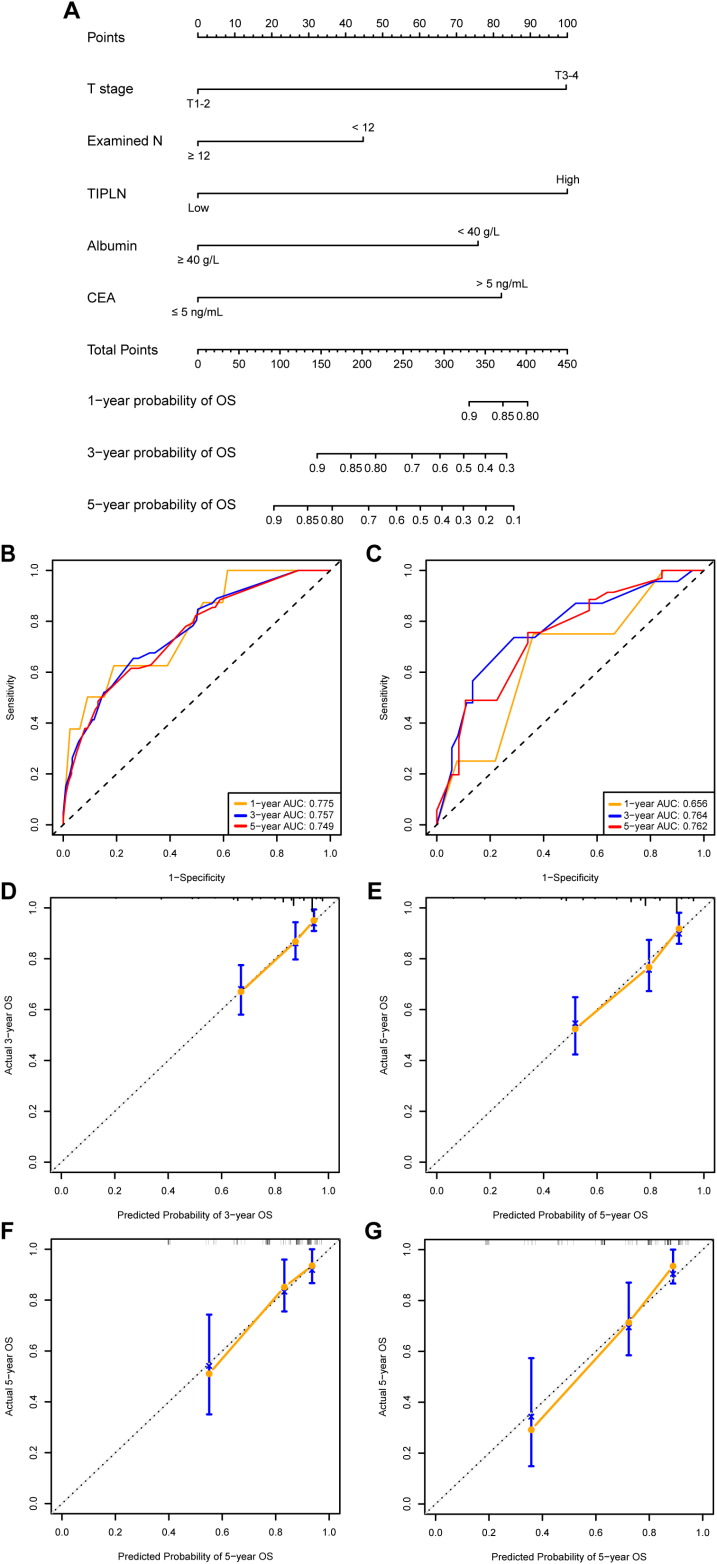
Construction and validation of prognostic models based on TIPLN. **(A)** Newly developed TIPLN-based nomogram. **(B, C)** AUCs of using the nomogram to predict OS probability in the training cohort **(B)** and validation cohorts **(C)**. **(D–G)** Calibration curves of 3-year and 5-year OS for N1 CRC patients in the training cohort. **(D, E)** and validation cohort **(F, G)**. Examined N, total number of lymph nodes examined; TIPLN, tumor infiltration proportion within lymph nodes; CEA, carcinoembryonic antigen; OS, overall survival; AUC, area under the receiver operating characteristic curve.

A TIPLN-based nomogram prognostic model was constructed using backward stepwise regression, incorporating TIPLN, T stage, Examined N, CEA, and albumin (**Figure 3A**). In the training cohort, the model demonstrated satisfactory predictive performance for 1-year, 3-year, and 5-year OS, with AUCs of 0.775, 0.757, and 0.749, respectively (**Figure 3B**). In the validation cohort, the AUCs for predicting 1-year, 3-year, and 5-year OS were 0.656, 0.764, and 0.762, respectively (**Figure 3C**). Furthermore, calibration curves showed good agreement between predicted and observed OS probabilities in both the training and validation cohorts (**Figures 3D–G**).

### Incremental prognostic value of TIPLN in the clinicopathological model

A clinicopathological nomogram was built based on multivariate Cox regression analyses without the TIPLN to elucidate the incremental value of TIPLN when integrated with clinicopathological variables for improving prognostic prediction (**Supplementary Table 1**, **Supplementary Figure 2**). The changes in C-index, NRI, and IDI were used to assess the accuracy of the clinicopathological nomogram and the TIPLN-based nomogram. In the training cohort, the C-index for the TIPLN-based nomogram was 0.739 (95% CI: 0.623-0.855) compared to 0.648 (95% CI: 0.513-0.783) for the clinicopathological nomogram. The NRI for 3- and 5-year OS were 0.180 (95% CI: 0.013-0.343, *P* = 0.031) and 0.436 (95% CI: 0.027-0.608, *P* < 0.001), respectively. Similarly, the IDI values for 3- and 5-year OS were 0.079 (95% CI: 0.032-0.147, *P* = 0.007) and 0.094 (95% CI: 0.041-0.175, *P* < 0.001) (**Table 3**). These results were validated in the validation cohort (**Table 3**), further supporting the superiority of the TIPLN-based nomogram.

**Table 3:** C-index, NRI, and IDI for the TIPLN-based nomogram vs. the clinicopathological nomogram in predicting survival in N1 colorectal cancer patients.

Index	Training cohort		Validation cohort	
	Estimate	95% CI	Estimate	95% CI
C-index				
TIPLN-based nomogram	0.739	0.623-0.855	0.753	0.575-0.930
Clinicopathological nomogram	0.648	0.513-0.783	0.682	0.518-0.846
NRI				
For 3-year OS	0.191	0.073-0.519	0.180	0.013-0.343
For 5-year OS	0.436	0.027-0.608	0.439	0.001-0.791
IDI				
For 3-year OS	0.079	0.032-0.147	0.078	0.007-0.203
For 5-year OS	0.094	0.041-0.175	0.098	0.019-0.248

NRI and IDI values are calculated vs. the clinicopathological nomogram.

TIPLN, tumor invasion proportion of lymph node; CI, confidence interval; OS, overall survival.

**Figure 4:**
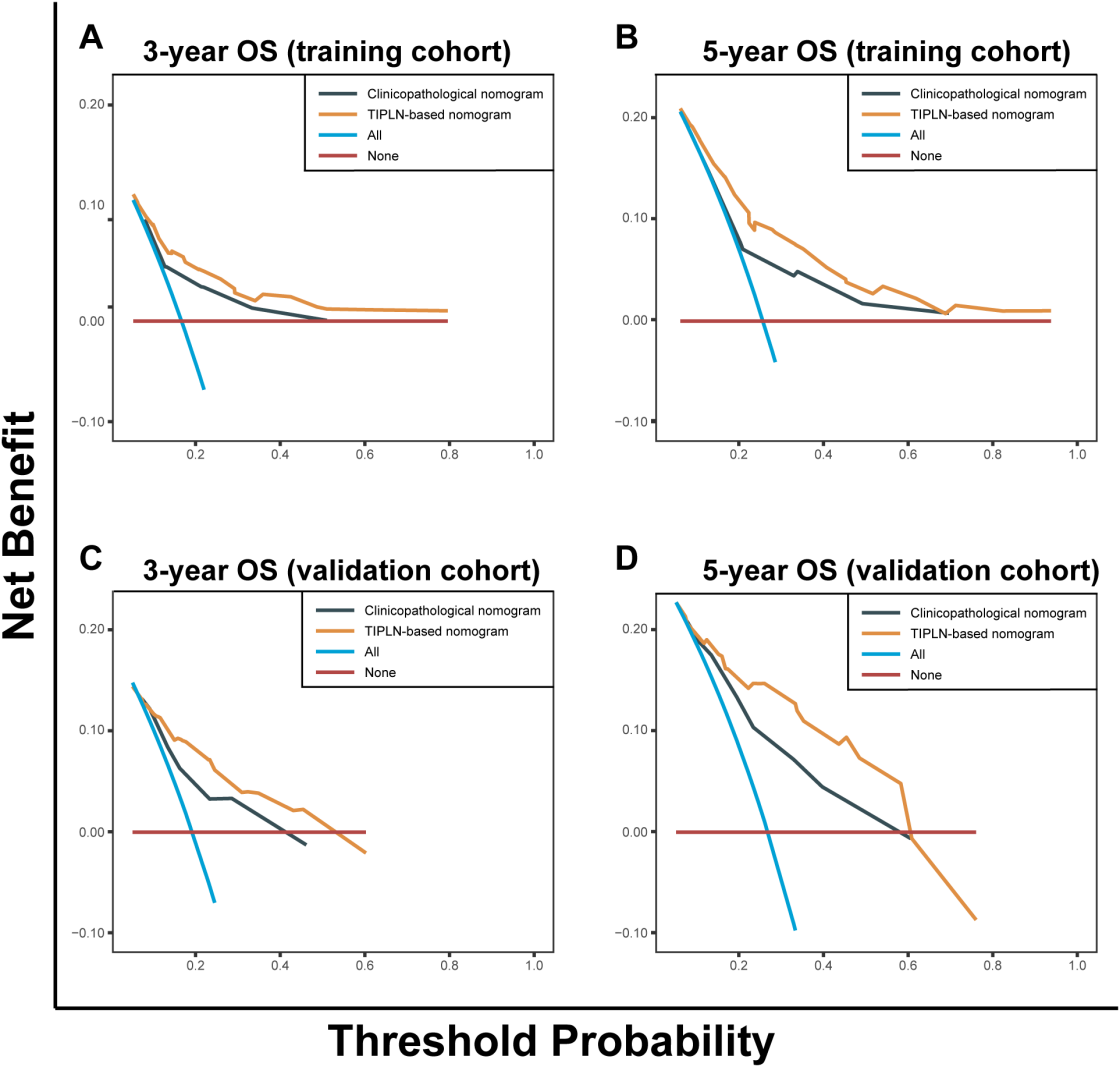
Decision curve analysis of the clinicopathological nomogram and TIPLN-based nomogram for the survival prediction of patients with N1 CRC. **(A)** 3-year survival benefit in the training cohort. **(B)** 5-year survival benefit in the training cohort. **(C)** 3-year survival benefit in the validation cohort. **(D)** 5-year survival benefit in the validation cohort. TIPLN, tumor infiltration proportion within lymph nodes; OS, overall survival.

The DCA demonstrated that the TIPLN-based nomogram provided superior predictions of 3- and 5-year OS, yielding greater net benefits compared to the clinicopathological nomogram across most threshold probabilities in both the training and validation cohorts. This superiority was consistent when compared to the treat-all and treat-none strategies, highlighting the clinical utility of the TIPLN-based nomogram in improving prognostic accuracy (**Figure 4**).

### Relationship between TIPLN and other lymph node indicators

We further investigated the relationship between TIPLN and other prognostic indicators, including LNR and LODDS. Our findings demonstrated a significant positive correlation between TIPLN and both LNR (**Supplementary Figure 3A**) and LODDS (**Supplementary Figure 3B**). These results suggest that TIPLN offers complementary prognostic information to enhance the predictive value of traditional indices.

## Discussion

In this study, we developed and validated a novel nomogram based on TIPLN for predicting the prognosis of patients with N1 CRC. Our findings demonstrated a significant association between high TIPLN levels and poorer outcomes identifying TIPLN as an independent and strong prognostic factor. The nomogram incorporated five variables selected through backward stepwise regression using the minimum AIC criterion, demonstrating robust discriminative ability and excellent calibration in both the training and validation cohorts.

The TNM staging system is a fundamental tool for determining prognosis and guiding individualized treatment strategies in CRC (8). However, survival rates can vary significantly, particularly among patients with stage III CRC (27). LN metastasis is recognized as a key prognostic factor in patients with non-distant metastatic CRC (28, 29). Despite its widespread adoption, the traditional N staging system has notable limitations. One major drawback is its reliance on the absolute number of positive LNs, which can be influenced by variability in the number of lymph nodes examined. This inconsistency can lead to inaccurate prognostic assessments.

Recent studies have explored alternative metrics, such as the LNR and LODDS, which consider both the number of positive LNs and Examined N (30–32). These metrics improve upon the limitations of the traditional N stage but remain primarily focused on the quantitative aspects of nodal involvement (33, 34). It has been demonstrated that a tissue 3D imaging technique can quantify the number of tumor cells within lymph nodes. The results suggest that tumor cells exhibit slow, gradual growth in the early stages, followed by rapid expansion in the post-adaptation phase, preparing them for distant metastasis (35). As tumors progress through their evolutionary course, they acquire the capacity for metastasis (36–38). LN involvement is a significant prognostic indicator, reflecting both the aggressive nature of tumor biology and serving as a potential mediator for subsequent distant metastasis (39). In line with these findings, we observed significant differences in TIPLN among positive LNs, underscoring the pathological heterogeneity of these nodes and their prognostic significance in colorectal cancer patients.

Interestingly, the prognostic value of TIPLN was still confirmed in multivariable model adjusting for other factors, such as T stage, Examined N, albumin, and tumor biomarkers. To further enhance clinical utility, the nomogram, which integrates TIPLN and other significant prognostic variables, demonstrated excellent accuracy in predicting 3-year and 5-year survival rates. The strong calibration and discriminative performance of the nomogram suggest its potential as a valuable tool for individualized prognosis assessment in clinical practice.

Despite these promising findings, this study has several limitations. First, as a single-center retrospective study, the findings may be subject to inherent biases and confounding factors, limiting generalizability. External validation in large-scale, multicenter cohorts including N2 patients, is necessary to confirm the prognostic value of TIPLN. Second, the anatomical distribution of LNs could not be systematically analyzed due to the absence of standardized spatial documentation in routine pathology records, thereby precluding adjustment for this potential confounder in prognostic assessments. Additionally, due to the retrospective nature of the study, progression-free survival data were not consistently available, limiting our ability to evaluate the association between TIPLN and disease progression. Furthermore, the influence of different chemotherapy regimens was not thoroughly evaluated. Future studies should include prospective, randomized controlled trials to further validate the robustness of TIPLN across different treatment modalities.

## Conclusions

Our study provides evidence that TIPLN is an independent prognostic factor in N1 CRC. The combination of TIPLN and other clinical variables is a strong predictor of patient prognosis, making it a valuable tool for individualized prognosis assessment and assisting in clinical decision-making.

## Data Availability

The original contributions presented in the study are included in the article/**Supplementary Material**. Further inquiries can be directed to the corresponding authors.
