# Supplementary material for: Prognostic and predictive value of tumor infiltration proportion within lymph nodes in N1 colorectal cancer
Source: Front Oncol. 2025 Mar 25;15:1512960. doi: 10.3389/fonc.2025.1512960 (PMC11975947; doi:10.3389/fonc.2025.1512960)
Supplement: Supplementary file 1 [file DataSheet1.docx]

***Supplementary Material***


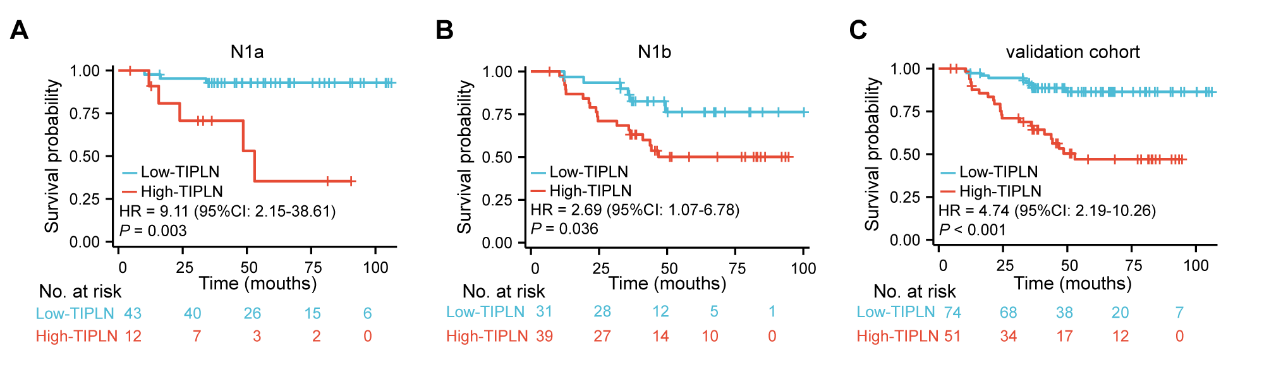


**Supplementary Figure 1. Kaplan-Meier survival curves stratified by TIPLN levels in the validation cohort of N1 CRC patients. (A)** Kaplan-Meier curve for N1a patients. **(B)** Kaplan-Meier curve for N1b patients. **(C)** Kaplan-Meier curve for the entire validation cohort. TIPLN: tumor infiltration proportion within lymph nodes; CRC: colorectal cancer HR: hazard ratio; CI: confidence interval.

**
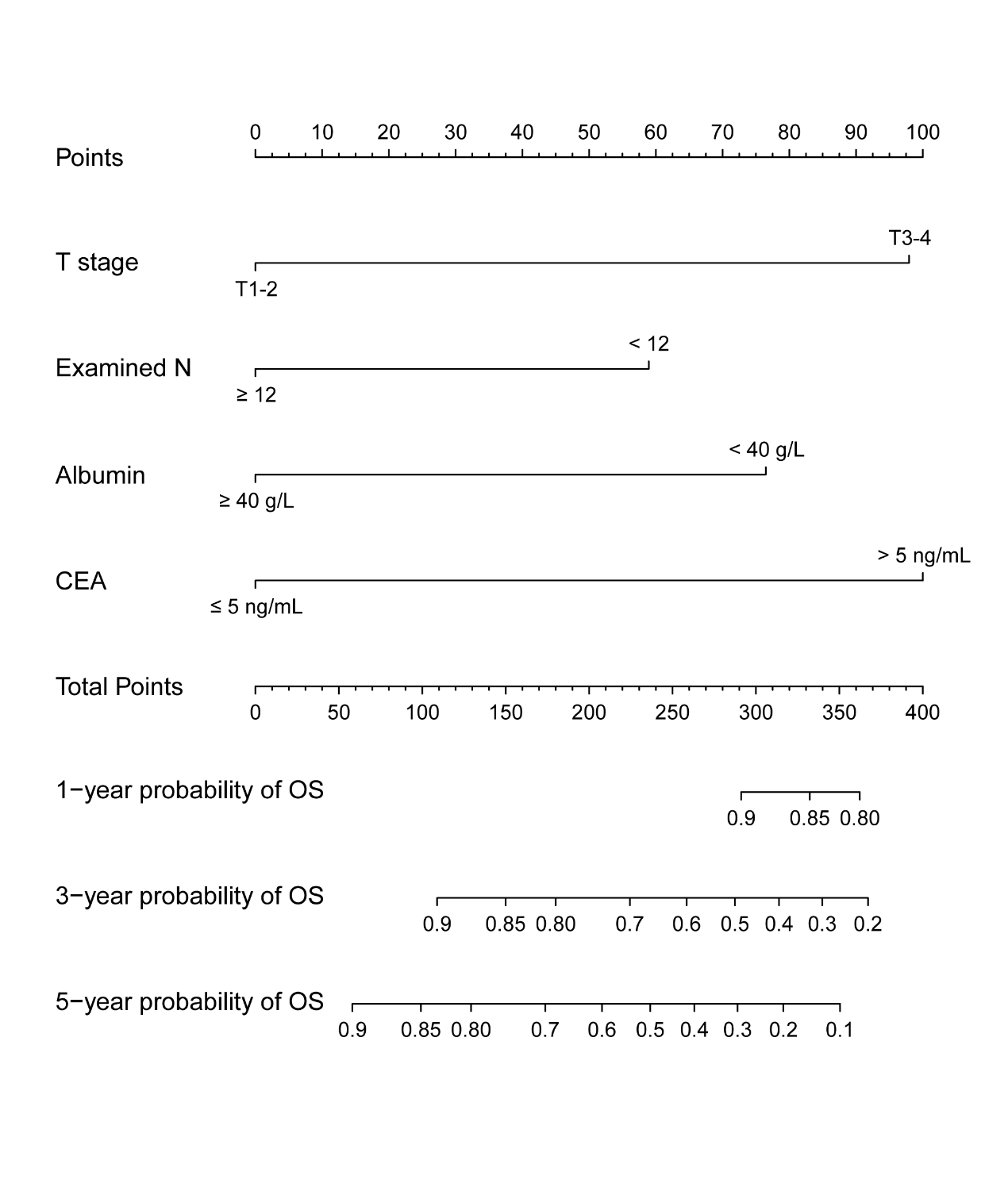
**

**Supplementary Figure 2. A clinicopathological nomogram for predicting OS in N1 CRC patients.** A nomogram developed to predict 1-, 3-, and 5-year OS probabilities in N1 CRC patients. Total points are calculated based on relevant clinical and pathological variables to determine the corresponding survival probabilities. OS: overall survival; CRC: colorectal cancer; Examined N: total number of lymph nodes examined; CEA: carcinoembryonic antigen.


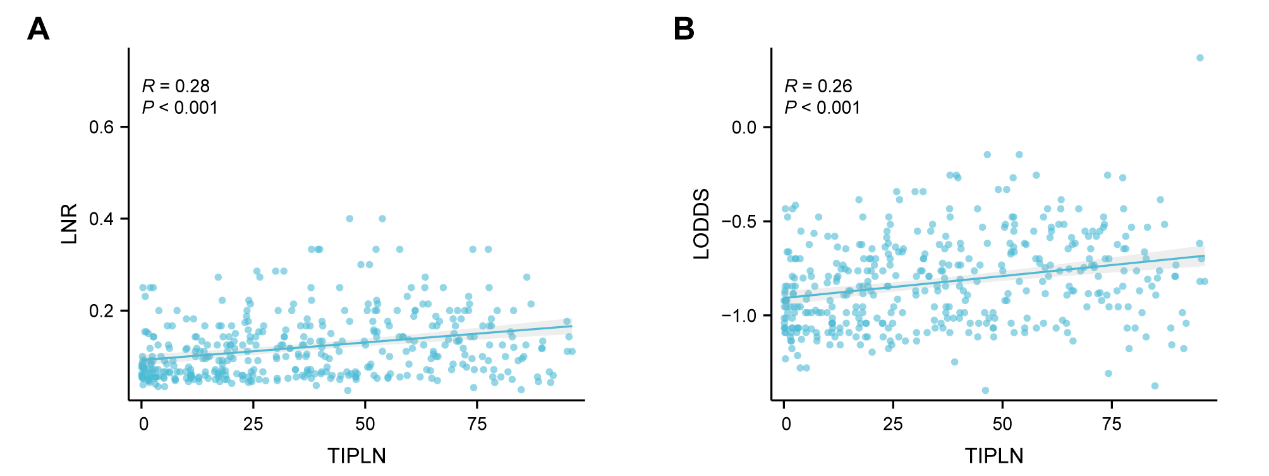


**Supplementary Figure 3. (A)** The relationship between TIPLN and LNR. **(B)** The relationship between TIPLN and LODDS. TIPLN: tumor infiltration proportion within lymph nodes; LNR: lymph node ratio; LODDS: log odds of positive lymph nodes.

**Supplementary Table 1. Multivariate analyses of overall without the TIPLN in the training cohort**

| **Variable** |  | **Multivariate analysis** | |
| --- | --- | --- | --- |
|  |  | **HR (95% CI)** | ***P*** |
| T stage |  |  |  |
| T1-2 |  | Reference |  |
| T3-4 |  | 2.68 (1.15-6.25) | 0.022 |
| Examined N  <12  1 (Reference)  1 (Reference)  ≥12 |  |  |  |
| ≥12 |  | Reference |  |
| <12 |  | 1.75 (0.98-3.12) | 0.058 |
| Albumin |  |  |  |
| ≥40 g/L |  | Reference |  |
| <40 g/L |  | 2.21 (1.35-3.62) | 0.002 |
| CEA |  |  |  |
| ≤5 ng/mL |  | Reference |  |
| >5 ng/mL |  | 2.31 (1.31-4.05) | 0.004 |
| CA19-9 |  |  |  |
| ≤37 U/mL |  | Reference |  |
| >37 U/mL |  | 1.55 (0.84-2.88) | 0.160 |
| CA125 |  |  |  |
| ≤35 U/mL |  | Reference |  |
| >35 U/mL |  | 1.96 (0.91-4.22) | 0.086 |

HR: hazard ratio; CI: confidence interval. Examined N: total number of lymph nodes examined; TIPLN: tumor invasion proportion of lymph nodes; CEA: carcinoembryonic antigen; CA19-9: carbohydrate antigen19-9; CA125: carbohydrate antigen125.
